# Perceived Barriers and Facilitators for Bedtime Routines in Families with Young Children

**DOI:** 10.3390/children8010050

**Published:** 2021-01-15

**Authors:** George Kitsaras, Michaela Goodwin, Michael Kelly, Iain Pretty, Julia Allan

**Affiliations:** 1 Dental Health Unit, Williams House, Manchester Science Park, The University of Manchester, Manchester M15 6SE, UK; michaela.goodwin@manchester.ac.uk (M.G.); iain.a.pretty@manchester.ac.uk (I.P.); 2 Department of Public Health and Primary Care, University of Cambridge, Cambridge CB2 1TN, UK; mk744@medschl.cam.ac.uk; 3 Institute of Applied Health Sciences, University of Aberdeen, Aberdeen AB24 3FX, UK; j.allan@abdn.ac.uk

**Keywords:** behavior change, child, sleep, parental, qualitative

## Abstract

Objectives: Bedtime routines are a highly recurrent family activity with important health, social and behavioural implications. This study examined perceived barriers to, and facilitators of, formulating, establishing, and maintaining optimal bedtime routines in families with young children. Design: Participants completed a semi-structured interview based on the Theoretical Domains Framework (TDF). Analysis followed a deductive approach. Participants: A total of 32 parents participated in the study. Most participants (*N* = 30) were females, were white (*N* = 25) and stay at home parents (*N* = 12). Results: Key barriers included lack of appropriate knowledge and sources of information, problematic skills development, social influences, cognitive overload, and lack of motivation for change. Facilitators included social role, access to resources, positive intentions, beliefs about consequences and reinforcement. In particular, optimal bedtime routines were less likely to be enacted when parents were tired/fatigued and there was a strong effect of habit, with suboptimal routines maintained over time due to past experiences and a lack of awareness about the importance of a good bedtime routine. Conclusions: Several theory-based, and potentially modifiable, determinants of optimal bedtime routines were identified in this study, providing important information for future interventions. Several of the key determinants identified were transient (tiredness) and/or non-conscious (habit), suggesting that future interventions may need to be deployed in real time, and should extend beyond conventional techniques.

## 1. Introduction

Bedtime routines are one of the most frequently performed family activities encompassing a series of actions undertaken by families with young children in the hour before bed [1] Most families start implementing their bedtime routines with their children from an early age [2]. Optimal routines should: (a) be consistent throughout the week and weekend following the recommended sleep times for each age group, (b) include tooth brushing, (c) avoid drinks (such as bottle feeding) and snacks the hour before bed, (d) minimise the use of electronic devices and television, and (e) include book reading and book sharing activities [3,4,5,6]. Other activities such as interactive play, bath before bed etc. can be considered as part of a family’s bedtime routine depending on its individual circumstances [2]. Past studies have demonstrated the importance of bedtime routines for both child-specific (quality of sleep (2), dental health [7], school performance and school readiness [5,8], BMI [9], psycho-social and emotional development [10] as well as parent/family-specific outcomes (overall family functioning and parental socio-emotional wellbeing) (2). Moreover, intervention studies have shown that it is possible to intervene and change routines with subsequent benefits for children and parents alike [2].

Bedtime routines are essentially a series of behaviours and therefore in order to change them we need to draw from the wealth of behaviour change literature. Existing studies and interventions on bedtime routines have utilised a wide range of theories and approaches (2). A widely used approach within behavioural science and behaviour change interventions is the Behaviour Change Wheel (BCW), a theory-informed and evidence-based guide to designing behaviour change interventions [11]. The BCW includes a stepped approach from the initial examination of a target behaviour to the development and implementation of an intervention designed to change and sustain a new behaviour [11]. Use of theory and a stepped, theory-informed and evidence-based approach, like the BCW, can increase the effectiveness and success of a subsequent intervention [11]. Also, the use of an established methodological approach (like the BCW) can allow for direct comparisons between work undertaken around bedtime routines and other repetitive, health-related behaviours.

In order to gain a better understanding of our target behaviour (bedtime routines) and enhance our ability to develop evidence-based interventions, the present study utilised a theoretical framework (the Theoretical Domains Framework—TDF). This evidence-based framework was used to explore the barriers and facilitators that parents from economically deprived households face when developing and maintaining bedtime routines for their children. The TDF is a framework which summarises 84 possible determinants of behaviour into overarching “theoretical domains”, allowing for a comprehensive exploration of all possible determinants of bedtime routines in families with young children [11]. The TDF has been extensively used in healthcare and behavioural research and it is described as the first and most critical step in the BCW (BCW) [11]. Through the use of the TDF, possible barriers and facilitators regarding bedtime routines can be systematically explored leading to greater understanding on this complex and highly recurrent behaviour and helping to identify potentially modifiable determinants of optimal and suboptimal bedtime routines.

### Objective

This qualitative study uses the TDF to explore perceived barriers and facilitators of the formation, establishment and maintenance of bedtime routines in families with young children.

## 2. Participants and Methods

### 2.1. Participants and Recruitment

A total of 32 parents with young children between the ages of 3–7 years old were recruited for this study. Parents were recruited in two distinct periods in 2018 and 2019. Parents were recruited from nurseries where a member of the research team approached parents directly providing details about the study and taking informed consent. A total of 40 parents were approached with 32 recruited (80% success rate). Inclusion criteria included: (a) ability to speak and comprehend English and (b) having a child between the ages of 3 and 7 years old. Participants were compensated for their time in the form of £10 vouchers for online shopping. All participants completed the interviews with no dropouts for this study. All elements of this study were approved by the University of Manchester Research Ethics Committee. The study in its entirety including consent forms and all study materials was previously approved by the Health Research Authority (Integrated Research Application System (IRAS) ID: 238552). All participants accepted anonymized use of their data for further analyses and subsequent publication during consent. Written consent was taken during recruitment.

### 2.2. Data Collection

Interviews were completed either in person or via telephone depending on participants’ preference. In total, 25 participants completed an in-person interview with 7 opting for a telephone interview. In each interview, a detailed semi-structured interview schedule based on the 14 TDF domains was used (Appendix A). Each TDF domain was explored with a combination of different questions. At the end of the interview, participants were encouraged to make additional comments and statements about elements of their own experience not covered by the interview schedule. Interviews lasted between on average 30 min and were all completed by the same interviewer. The interviewer had extensive experience in conducting qualitative work and has worked previously with the TDF framework. To ensure adequate content validity the interview schedule was based on published TDF schedules-approach and it was reviewed and refined by an expert.

### 2.3. Data Analysis

Each audio recording was transcribed verbatim using a transcription service. Two independent coders with experience in using the TDF framework used a deductive approach to map each statement to one of the TDF domains (or code as outside of the TDF). Any disagreements in coding were resolved through discussion. Remaining disagreements were resolved by a third independent, experienced coder. Barriers and facilitators were identified based on participants’ responses. Overarching themes were also identified and summarized while frequency counts were used to determine the most commonly endorsed domains and specific component constructs.

## 3. Results

### 3.1. Sample Characteristics

In total, 32 individuals (30 females and 2 males, aged 35 (SD = 3) participated in the study. The majority of the participants were white (*N* = 25), three participants of Asian/British-Asian ethnic background and four participants of Black/Black-British/Caribbean ethnic background. In terms of educational level, *N* = 12 had University education, *N* = 20 were college graduates (either having completed their A-levels (*N* = 10) or high school graduates (*N* = 10)). The majority of participants had either one (*N* = 15) or two children (*N* = 15) with only two participants having three children. Most participants were stay at home parents (*N* = 12), with *N* = 10 participants working part-time, *N* = 8 participant working full-time and *N* = 2 participants studying at University. The majority of participants (*N* = 27) were in two parent households, a further three were in single parent households and two participants came from households with more than two adults (multigenerational households with at least one grandparent present in the household). Finally, in terms of deprivation as calculated by the Index of Multiple Deprivation (IMD) most participants (*N* = 20) were on the fifth quintile (most deprived). *N* = 6 were on the fourth quintile with only six participants on the third quintile. Average IMD score was 36.4 (SD = 4.1) classifying as “most deprived”. The IMD is a frequently used metric of social deprivation in England (National Perinatal Epidemiology Unit, University of Oxford) and it provides data based on participants’ postcodes.

### 3.2. Inter-Rater Reliability

Cohen’s kappa (κ) was calculated in order to examine inter-rater reliability between the two independent coders. A total of 289 statements were examined and mapped into relevant TDF domains. Based on the results of the analysis and following guideline outlined by Landis and Koch (1977) there was substantial agreement between the two coders (*κ* = 0.891, *p* < 0.005).

### 3.3. Barriers and Facilitators by TDF Domain

Example quotations are presented below per each TDF domain to showcase participants’ replies.

#### 3.3.1. Knowledge

In general, all parents reported awareness of the importance of bedtime routines. Most parents were able to describe what a good bedtime routine should look like. Use of electronics before bed was the most common activity that parents did not mention when describing a good bedtime routine. Table 1 summarises participants’ views on what constitutes an optimal routine. The vast majority (*N* = 25) of parents reported that they had never been offered advice on bedtime routines when their children were born.

“Reading that the school asks us to do. Spellings and settling them down in a relaxed environment before bedtime and teaching them that it’s healthy to look after their teeth and that is one of the bedtime routines that, as they get older, that they should be doing.”

(QI021)

“No. It would have been good to get some advice, but no one really said anything about routines when the children were born.”

(QI012)

Six participants knew about official recommendations or were given some advice when their children were younger, but they could not recall exactly what they were told or who provided them with that information. A total of 15 participants expressed a positive view about how useful an official system or point of contact where they could seek advice on bedtime routines would have been.

“If somebody could have told me how to get my kids to sleep that probably would have been really, really helpful.”

(QI005)

#### 3.3.2. Skills

In terms of skills development, most parents reported using the same sort of routines with their own children as they had when they were children. While some parents mentioned external factors that influenced the development of their bedtime routines and most parents were able to identify a variety of skills and techniques they use as part of their bedtime routines.

“So when they’re doing their teeth, we have, like, one of their favourite songs will play … so they’ll find a song that’s three and a half minutes long, so they’ve got to brush their teeth while that song is playing, so they’ll dance while they’re brushing their teeth and then once that songs finished, their teeth are done.”

(QI004)

#### 3.3.3. Social/Professional Role/Identity

Parents viewed themselves as an important role model for their children and felt a huge level of responsibility for the overall wellbeing and development of their children. Some parents brought their overall, non-parental, roles and identities as professionals in the context of their responsibility towards their children.

“I suppose what I’m doing as a parent is trying to set them up in good habits for the rest of their lives, because the stuff that they do before they go to bed is the stuff that I do before I go to bed.”

(QI025)

#### 3.3.4. Beliefs about Capabilities

A total of seven parents stated their bedtime routines were generally not perceived to be difficult or challenging. However, parents identified some occasions when routines were perceived as more challenging, for example over the weekend. Ten parents felt that their bedtime routines are difficult and challenging in general.

“Difficult but it’s something that we’re all used to, and they’ve done since they were younger and it’s something, like I say, that I’ve always been consistent with but yes, it is difficult.”

(QI031)

#### 3.3.5. Optimism

The majority of parents appeared confident and optimistic about how things will unfold in the future regarding their bedtime routines.

“I don’t feel anxious about it really, I think... I haven’t really thought about it really. I don’t know.”

(QI005)

#### 3.3.6. Intentions

The majority of parents stated clear intentions to try actively to achieve and maintain good bedtime routines for their children in the short and long-term future.

“Yes, I mean 100 percent, 101 percent really and in terms of that, it’s maintaining and being consistent and that can get tiring but that’s the length that I personally am happy to go to for them.”

(QI011)

#### 3.3.7. Beliefs about Consequences

Most parents mentioned specific outcome expectations associated with problematic bedtime routines. While others reported their overall beliefs about the future of their children and the importance of having a good bedtime routine.

“If you’re brushing your teeth so this will give you a future with nothing a problem with your teeth and everything. But if you do brush correctly, in the correct way.”

(QI023)

#### 3.3.8. Reinforcement

Reinforcement was analyzed in two contexts: (a) reinforcement used towards the children as part of the bedtime routine or general parenting and (b) reinforcement experienced by the parents at the end of the night and after the children were off to bed. In terms of reinforcement techniques used with the children, most parents were able to list several techniques covering both positive (reward) and negative (punishment) reinforcement. In terms of reinforcement experienced by the parents, this included immediate benefits of having an optimal routine in place such as free time at the end of a busy day.

“It is a nice feeling, if it’s all smooth and everybody goes to bed happy and so on and you don’t feel like you’re on your last nerve, then, yes, of course it’s a nice feeling, because then you look at these two sleeping angels and think that’s lovely.”

(QI005)

#### 3.3.9. Goals

For many of parents, bedtime routines are more than just getting the children to bed, it is about spending good, quality time together and building long-lasting memories. Also, parents gave examples of goal priorities shifting when dealing with changing circumstances in their houses during their bedtime routines.

“You’re all busy during the day, the children are at school, you’re at work, so that is a really nice time to talk to the children and find out what’s been going on in their day and yes, they play with each other, it’s their time as well to have a bit of fun with each other.”

(QI011)

#### 3.3.10. Emotions

Parents reported a mixed emotional reaction to bedtime routines with some reporting negative emotional reactions towards them.

“Calm, quite fine, like I say because we’ve stuck to the same routine. It’s not a chore; it’s a pleasurable thing to do.”

(QI008)

#### 3.3.11. Memory, Attention, and Decision Process

All parents reported a high level of automation (memory) when it comes to their bedtime routines with little to no thought on what to do and how to do it. However, when tired (cognitive overload), parents reported difficulties in complying with their normal routine as well as issues around forgetting what they need to do.

“God, yes, it’s hard, well it can be just because I work full time and by the end of the day I’m shattered so, yeah, because they’re busy and they’re five and three.”

(QI023)

#### 3.3.12. Environment and Resources

Houses and the immediate environmental context did not present as an issue. All parents reported adequate access to all required resources (i.e., books, toothbrushes, tooth paste etc.) for achieving a good bedtime routine.

“Well the children have to share a room which makes things more difficult. It would have been nice to be able to have separate rooms for them but that’s not a possibility unfortunately.”

(QI012)

#### 3.3.13. Social Influences

Peer support (social support) was important for 15 parents in this study especially due to lack of any other available source of information. Meanwhile, 15 parents reported comparing their routines to their peers (social comparisons) with some of them expressing beliefs on whose routine is better and why.

“Yes, they always resist, every night they resist at bedtime and obviously at the weekends, I’m a little bit more lenient but no I think they enjoy the bedtime routine.”

(QI011)

#### 3.3.14. Behavioural Regulation

In terms of self-monitoring, a total of 14 parents reported not using any type of self-monitoring with regards to their bedtime routines reflecting the automated, habitual nature of the routines. However, 15 parents reported using specific self-monitoring techniques. Some parents reported specific habit breaking events that led to a significant change of behaviour in the past.

“Just in my head and keeping track of how it works well for the children and varying it upon that.”

(QI007)

### 3.4. Overarching Themes

Across the whole dataset, overarching themes, or factors that emerged as most important in relation to bedtime routines included: (a) lack of provision of information from respected sources, especially when children were younger and routines were being developed; (b) skills development and social support through peers; (c) parents’ beliefs that looking after their children’s bedtime routines is part of their parental role, their responsibility; (d) parents’ self-confidence and the emotional reactions associated with bedtime routines; (e) optimism about the future with clearly defined intentions to achieve and maintain good routines for their children; (f) positive reinforcement from good bedtime routines and negative reinforcement from bad bedtime routines; and (g) the level of automation and self-monitoring during bedtime routines.

### 3.5. Barriers and Facilitators

The key barriers and facilitators identified regarding formation, establishment and maintenance of optimal bedtime routines are summarised in Table 2 below.

## 4. Discussion

This study examined perceived barriers to, and facilitators of, the formation, establishment, and maintenance of bedtime routines using the TDF. The examination of barriers and facilitators is a vital step for the development of theory-informed and evidence-based interventions to support and assist parents with their bedtime routines.

**Figure 1:**
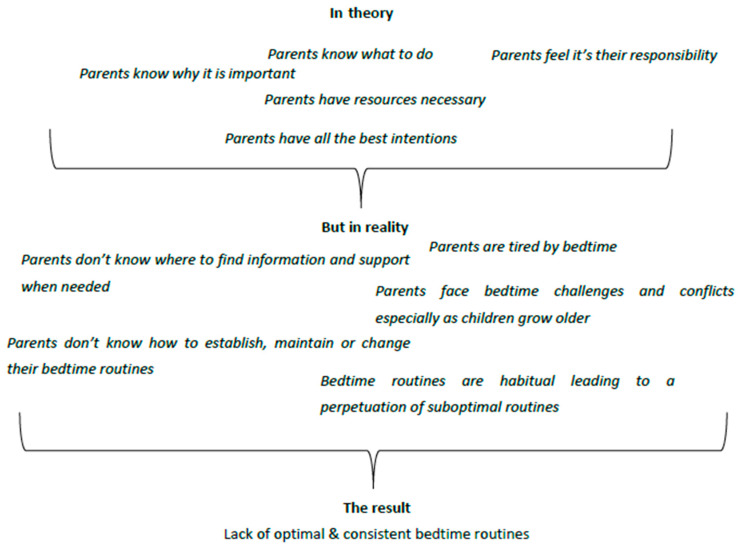
Schematic representation of key barriers and facilitators based on TDF. Flowchart presenting key barriers and facilitators identified by the study leading to the creation of sub-optimal bedtime routines.

In line with recent studies in this area [12] it is evident that many of the important ingredients required to establish and maintain optimal bedtime routines are in place: (a) parents are aware of why they need optimal bedtime routines, (b) they know what they have to do as part of an optimal routine, (c) they have the resources required, (d) they recognize the benefits of achieving good routines for themselves as well as for their children, (e) hold intentions to achieve them, and (f) feel that it is their responsibility as parents to provide consistent and beneficial routines to their children. In contrast, problems in achieving optimal bedtime routines arise when: (a) parents are tired; (b) children present with more challenging behaviours bringing social comparisons and conflicts into the family unit; (c) parents seek but are unable to find information on how to change (or establish) their bedtime routines due to the lack of clearly marked, official sources of information; (d) parents heavily rely on past experiences; (e) parents feel that routines are a habit that they cannot change; and (f) parents feel unmotivated to change. Figure 1 provides a visual schema for the key outcomes.

Past actions are a strong predictor of future behaviours and, people tend to stick with their behaviours unless they prove to be problematic early on [13,14,15,16]. Habitual behaviours in stable contexts (like bedtime routines) have higher likelihood of being reflecting past behaviours and experiences [15,17]. This likelihood increases even further when little to no consideration, reflection, or self-monitoring is in place to critically appraise past experiences and behaviours and their influence on current behaviours [13]. Biased scanning theory and self-perception [18] theory suggest that when people engage in a particular behaviour (for example, when establishing their routines) they often conduct “a biased search of memory for previously acquired knowledge that confirms the legitimacy of their actions” with “with little if any conscious deliberation, simply reasoning that if they have performed the behaviour voluntarily, they must consider it to be desirable” [19] In the context of bedtime routines, parents might behave in a certain way that in their own opinion reflects an optimal bedtime routine based on their past experiences (heuristic behaviour) with little reflection (self-perception theory) and a biased justification for their actions (biased scanning theory). In this study, lack of appropriate provision and sources of information available to parents (especially first-time parents), appears to lead to a heavy reliance on past experiences for information about what constitutes an appropriate bedtime routine. This is then habituated with little self-monitoring and may hinder parents’ ability to formulate and maintain optimal routines.

Parental tiredness/fatigue and cognitive overload acted as additional barriers to systematically and consistently achieving optimal bedtime routines even when parents knew what they needed to do and how to do it. In general, parental tiredness is a nearly universal experience [20]. Multiple child and non-child related factors contribute to parental fatigue [21]. The demands of infant and toddler care combined with domestic and professional workload as well as other responsibilities result in significant levels of tiredness and fatigue for parents [20] Fatigue is closely associated with parental wellbeing, parental self-efficacy, parental anxiety, parental mood, low warmth, and irritability during parent–child interactions resulting in suboptimal parenting with less engagement in shared parent–child activities [20,22]. In turn, these parental difficulties and problematic parent–child interactions can result in a range of child emotional and behavioural difficulties later in life [23]. Bedtime routines due to their highly recurrent nature and the particular time of the day that they need to be implemented are particularly vulnerable to the effects of tiredness and fatigue. Addressing the effects of parental tiredness and fatigue during bedtime routines is not an easy task especially since parental fatigue is caused by a combination of factors.

Finally, lack of motivation, negative emotions and automation of routines are another set of barriers identified in this study. These barriers can be grouped under the term “behavioural inertia” [24]. Behavioural inertia is a term commonly used in behavioural economics and it is associated with inaction and a tendency to remain with the status quo [24]. When faced with a decision, individuals tend to prefer the status quo since it provides them with comfort and a sense of familiarity [25]. This preference for the status quo fuels a lack of motivation which in return maintains the status. Fear of change and fear of the unknown, of the possible alternatives if pursuing a different pathway is another important factor that fuels the status quo bias and behavioural inertia [26].

**Figure 2:**
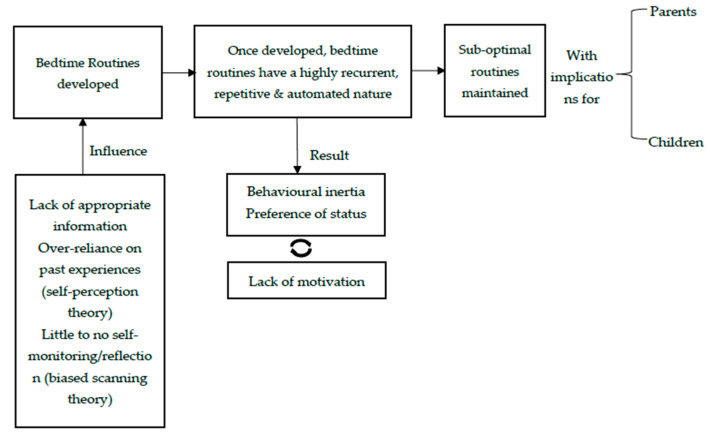
A proposed mechanism for the initial creation and later maintenance of sub-optimal bedtime routines based on TDF results on ‘barriers and facilitators’ (Table 2). Sleep is a vital part of health and wellbeing with multiple and wide ranging implications for child development and wellbeing [2]. Sleep plays a major role for children’s development with poor sleep hygiene and sleeping habits associated with a series of problematic outcomes across physical health [27], neurocognitive development [28], socio-emotional development [29] and family functioning [30]. The importance of bedtime routines for sleep has been recently highlighted by a recommendation by the American Academy of Pediatrics (AAP) which issued a series of sleep health recommendation including the need of a consistent bedtime routine and childhood routines in general [2]. Apart from quality of sleep, sub-optimal bedtime routines could affect a series of other areas including dental health [6], school readiness and school performance [5,8] and BMI [9].

Behavioural inertia and status quo bias in the right context can be useful in maintaining optimal behaviours however, in cases where change would be beneficial they transform to detrimental factors perpetuating problematic behaviours [25]. For bedtime routines, behavioural inertia is manifested in the lack of motivation and automation of routines from the parent’s perspective. Routines develop when children are in their infancy but fairly quickly, bedtime routines show signs of stability with most activities in place. If a family is lacking an optimal routine at this early stage, then the automated, highly recurrent and repetitive nature of bedtime routines overtakes the need or sense of urgency for altering and improving them. The end result is a self-perpetuating cycle where change is not considered as a realistic prospect. Figure 2 provides an overview of the way these factors could potentially contribute to the formulation and maintenance of sub-optimal bedtime routines.

## 5. Limitations

The development of the interview schedule to reflect and capture all TDF domains might have resulted in a more rigid and structured discussion. However, this particular possible limitation was managed through the establishment of prior good rapport that allowed for participants to feel more comfortable and express themselves in their own way. Participants were also given the freedom to discuss anything outside of the topic guide that they felt was relevant to any aspect of bedtime routines. Also, the disproportionate number of white and mainly female participants, while problematic for generalization of results, it is not surprising given this particular area of research and the context of recruitment. Also, the household composition of our participants, mainly two parent households, limits generalizability in more diverse household types that might be more relevant and prevalent in other countries and/or specific areas. Finally, another possible limitation can be found in the selection of participations using a convenience sampling approach that could have resulted in biases around demographic and socio-economic characteristics.

## 6. Implications for Further Research

Using an evidence-based approach there is a possibility to map identified barriers and facilitators into existing literature and evidence around behaviour change and techniques. Those techniques could either maintain and promote facilitators (i.e., motivation to achieve optimal routines, knowledge around importance of routines, etc.) while removing barriers (i.e., tiredness and cognitive overload through simple and easy to remember and implement techniques, provision of information when necessary, etc.). With some crucial barriers clearly identified, future work will focus on how to address them using a theory-informed and evidence-based approach that can maximise the effectiveness of subsequent intervention.

## 7. Conclusions

Parents of young children face a series of barriers to achieving optimal bedtime routines ranging from lack of appropriate knowledge to lack of motivation and tiredness. These barriers can prove detrimental for bedtime routines with possible health, behavioural, and social consequences for parents and children. Gaining a better understanding of the determinants of optimal and suboptimal bedtime routines, is an important first step for future more in-depth examinations and potentially intervention studies.

## Figures and Tables

**Table 1:** “In your opinion, what should a good bedtime routine include?”

ID	Consistency	Tooth Brushing	Avoiding Snacks/Food	Use of Electronics	Reading Before	Winding Down
QI001		X	X	X	X	
QI002	X	X	X	X	X	X
QI003		X	X		X	
QI004		X			X	
QI005	X	X	X		X	X
QI006	X	X	X		X	
QI007	X	X	X		X	X
QI008	X	X	X	X	X	X
QI009	X	X	X	X	X	X
QI010	X	X			X	X
QI011	X	X	X		X	X
QI012	X	X	X		X	X
QI013		X	X	X	X	
QI014	X	X	X	X	X	X
QI015		X	X		X	
QI016		X			X	
QI017	X	X	X		X	X
QI018	X	X	X		X	
QI019	X	X	X		X	X
QI020	X	X	X	X	X	X
QI021	X	X	X	X	X	X
QI022	X	X			X	X
QI023	X	X	X		X	X
QI024	X	X	X		X	X
QI025	X	X	X	X	X	X
QI026	X	X			X	X
QI027	X	X	X		X	X
QI028	X	X	X		X	X
QI029		X	X	X	X	
QI030	X	X	X	X	X	X
QI031		X	X		X	
QI032	X	X	X	X	X	X

X shows that activity was considered important as part of a bedtime routine.

**Table 2:** Key barriers and facilitators identified

Barrier	Facilitator
**‘Knowledge’** and **‘skills development’** were two of the most important barriers identified: (a) The majority of parents relied primarily on what their own parents used to do when they were children. If a parent had a bad bedtime routine as a child and with no alternative information on bedtime routines available some parents were left unable to recognize what is right and what is wrong with regards to bedtime routines and most importantly, how to change their routines in a meaningful way. (b) Seeking information online or relying on peer support was a mechanism that some parents deployed however, for some that was not possible and the quality and trustworthiness of information might not be consistent and appropriate for all cases. (c) Parents seemed unaware of where/who to approach should any issues with their bedtime routine arise or when their children are first born leaving them exposed to a potentially problematic start with their bedtime routines.	***Beliefs about consequences*** and the realization from many parents that bedtime routines can have a long-term effect to their children’s overall wellbeing and development was an important facilitator. Awareness of consequences when combined with clearly stated intentions and strong beliefs about the parental role and responsibility can be a powerful combination that can ultimately help parents to achieve and maintain optimal bedtime routines.
**‘Social influences’** and **‘intergroup conflicts’**—Within the family unit the interactions between parents and children were another important barrier for implementing good bedtime routines. The older the children, the more exposed to peer pressure and outside points of view resulting in higher frequency of arguments and conflicts within the family unit and at bedtime.	***Social role*** was an important facilitator for parents who wanted to provide their children with the best chances in life through, in part, their bedtime routines. Parents expressed deep and strong beliefs about the importance of their role in their children’s overall development and future wellbeing.
**‘Tiredness’** or **“cognitive overload”** was a significant barrier for achieving good bedtime routines especially in families with more than one child or families where the mother, as the one who’s primarily involved in bedtime routines, was working full-time.	***Environment and access to resources*** were both important facilitators for establishing and maintaining optimal bedtime routines since almost all families did not consider them as an issue. Also, all families mentioned that they are able to access all required resources in terms of toothpaste, tooth brushes, books, etc. that form part of an optimal routine.
**‘Habituation’** and **‘lack of self-monitoring of routines’** can act as barrier. Most parents reported just doing their bedtime routines as a habit with little thought. Habits are not by definition bad. It depends on what exactly the habit entails. Habits may serve to maintain and perpetuate good routines over time. However, if the bedtime routines of a family are sub-optimal, habituation of that routine with no self-reflection or time to actively think about the routine can result in a vicious cycle with the same, unhelpful and potentially harmful behaviours repeated every night.	***Intention*** is an important facilitator since almost all parents stated clear intentions to be able to have and maintain good bedtime routines for their children and especially as the children are growing older.
**‘Lack of motivation’** and **‘negative emotions’** associated with bedtime routines are an important barrier that contribute to parents feeling incapable of achieving optimal routines in a consistent manner or making positive changes to their bedtime routines where required.	***Reinforcement*** at the end of each night, depending how the routine unfolded, can be important facilitator for achieving, and especially for maintaining, good bedtime routines.

## Data Availability

The data presented in this study are available on request from the corresponding author. The data are not publicly available due to the conditions attached to the ethical approval granted for this study.

## Appendix A. Interview Schedule Based on the Theoretical Domains Framework

**Interview Schedule**

0 **Introduction**
  0.1. Briefly discuss the scope of this interview as an additional source of information in trying to get a better understanding of bedtime routines in families with young children. Discuss overall set up, including time, audio recordings, etc.
1 **Bedtime routines overview**
  1.1. Can you describe your typical bedtime routine? What time does it start and end? What does it involve? In what order? Who’s involved?
2 **General knowledge and skills**
  2.1. (KNOWLEDGE) How do you think a good bedtime routine looks like? What would be involved?
  2.2. (KNOWLEDGE) You’ve mentioned X, Y, and Z as things that would be involved in a ‘good’ bedtime routine—why do you think they’re important/why do they matter?
  2.3. (KNOWLEDGE) Are you aware of guidelines/recommendations relating to bedtime routines?
  If yes, what do they usually include? If yes, who/how did you come across those guidelines/recommendations? (Hint: midwives, health visitors, internet, etc.)
  Following reply to Qs.1 and 2.1/2.2/2.3, provide a quick definition of what an optimal bedtime routine should include and use a visual aid to quickly refer to with regards to all four main components (tooth brushing, book reading, diet, and use of electronics).
  2.4. (SKILLS) What skills do you think you would need in order to be able to do things involved in a good bedtime routine? (point to prompt card)
  2.5. (SKILLS) Which of these skills do you think you have? Are there ones you could do with improving?
  2.6. (SOCIAL IDENTITY) Who is responsible for bedtime routines in your view? (***Hint***: me as parent, parents or others?)
  2.7. (SOCIAL IDENTITY) Do you think other parents have good bedtime routines? Are they like you?
  2.8. (SOCIAL INFLUENCES) (If someone’s involved) How do you feel about your partner’s/husband’s/wife’s etc. involvement in your bedtime routines? Do they help or hinder your activities? (If no one’s involved) Do you wish there was someone to help you with your bedtime routines? In what way someone else could be helpful for you during your bedtime routines?
  2.9. (SOCIAL INFLUENCES) What do your friends/family think about your bedtime routine? Do you care what they think?
  2.10. (SOCIAL INFLUENCES) What do your kids think about the different bits of this good bedtime routine (Hint: prompt card)? How important is it to you what they think about it?
3 **Current situation**
  3.1. (BELIEFS CAPABILITIES) How easy or difficult is it for you to do your bedtime routine every night? Can you manage even when things are difficult?
  3.2. (BELIEFS CAPABILITIES) How confident are you in completing your bedtime routine every night? (If confidence low) What would make you feel more confident? (If confidence high) What gives you confidence?
  3.3. (EMOTIONS) How do you feel when you do manage to do all the things involved in your bedtime routine? What about when you don’t? What words describe how you typically feel during your bedtime routines (i.e., stressed, calm, happy, sad)?
  3.4. (EMOTIONS) How does the way you feel as it’s coming up to bedtime influence whether or not you do the things involved in a good routine?
  3.5. (MEMORY, ATTENTION & DECISION PROCESS) Are these different things (prompt card) things you do routinely, without thinking? Do they happen at a set time and in the same way every night or do you actively have to remember to do them?
  3.6. (MEMORY, ATTENTION & DECISION PROCESS) Do your routines change between school days and weekends? If so, how are they different? Do you still complete all of your activities?
  3.7. (MEMORY, ATTENTION & DECISION PROCESS) When you’re tired or have a lot on your mind, do you try and complete all activities? If not, how do you decide which activities to leave out?
  3.8. (ENVIRONMENT-RESOURCES) (If employed) Do you believe that your work affects your bedtime routines? If yes, in what way?
  3.9. (ENVIRONMENT-RESOURCES) Are there things about your home (e.g., where the bedrooms are, need to share rooms, noise, etc.) that make your bedtime routines easier or harder?
  3.10. (ENVIRONMENT-RESOURCES) Do you have access to the things you need to do the 4 things outlined here (prompt card), e.g., selection of books, toothbrushes, water/milk, etc.? If not, in what way?
  3.11. (REINFORCEMENT) When you have a good bedtime routine, what happens and how do you feel straight afterwards? What were the immediate benefits?
  3.12. (REINFORCEMENT) When you have a bad bedtime routine, what happens and how do you feel straight afterwards? What were the immediate consequences?
  3.13. (GOALS) What is/are your end goal(s) when starting your bedtime routines on a nightly basis?
  3.14. (GOALS) Compared to other things you need/want to get done, how important is it that you do all the things listed as part of your bedtime routine?
  3.15. (BEHAVIOUR REGULATION) Do you monitor your bedtime routines in any way? If yes, how do you do it? If not, do you believe that it might be useful to monitor them?
  3.16. (BEHAVIOUR REGULATION) (If monitor bedtime routines) What do you do when you notice that your bedtime routines are not as good as they used to be? Do you take any actions? If yes, what?
4 **Looking ahead**
  4.1. (BELIEFS CONSEQUENCES) Looking ahead in the future, what do you think will happen if you have a good bedtime routine in place? For you, your child, your family or in general? What will happen if you don’t? Do some of these things (prompt card) matter more than other for the future?
  4.2. (BELIEFS CONSEQUENCES) Do the future benefits outweigh the costs? How?
  4.3. (OPTIMISM) Looking ahead, how do you feel about your upcoming bedtime routines for the days, weeks, and years to come?
  4.4. (OPTIMISM) Do you feel that regardless of what happens day to day, things will turn out fine in the end?
  4.5. (INTENTIONS) Do you want to have a good bedtime routine? If yes, to what extent?
  4.6. Do you feel ready and able to make any changes that are necessary to your existing bedtime routine?
